# Racial and ethnic disparities in COVID-19 hospital cost of care

**DOI:** 10.1371/journal.pone.0309159

**Published:** 2024-10-14

**Authors:** Tricia J. Johnson, Joshua Longcoy, Sumihiro Suzuki, Zeynep Isgor, Elizabeth B. Lynch

**Affiliations:** 1 Department of Health Systems Management, Rush University, Chicago, IL, United States of America; 2 RUSH BMO Institute for Health Equity, Rush University, Chicago, IL, United States of America; 3 Department of Family and Preventive Medicine Rush University Medical Center, Chicago, IL, United States of America; UT Southwestern: The University of Texas Southwestern Medical Center, UNITED STATES OF AMERICA

## Abstract

**Introduction:**

Although studies have evaluated the hospital cost of care associated with treating patients with COVID-19, there are no studies that compare the hospital cost of care among racial and ethnic groups based on detailed cost accounting data. The aims of this study were to provide a detailed description of the hospital costs of COVID-19 based on individual resources during the hospital stay and standardized costs that do not rely on inflation adjustment and evaluate the extent to which hospital total cost of care for patients with COVID-19 differs by race and ethnicity.

**Methods:**

This study used electronic medical record data from an urban academic medical center in Chicago, Illinois USA. Hospital cost of care was calculated using accounting data representing the cost of the resources used to the hospital (i.e., cost to the hospital, not payments). A multivariable generalized linear model with a log link function and inverse gaussian distribution family was used to calculate the average marginal effect (AME) for Black, White, and Hispanic patients. A second regression model further compared Hispanic patients by preferred language (English versus Spanish).

**Results:**

In our sample of 1,853 patients, the average adjusted cost of care was significantly lower for Black compared to White patients (AME = -$5,606; 95% confidence interval (CI), -$10,711 to -$501), and Hispanic patients had higher cost of care compared to White patients (AME = $8,539, 95% CI, $3,963 to $13,115). In addition, Hispanic patients who preferred Spanish had significantly higher cost than Hispanic patients who preferred English (AME = $11,866; 95% CI $5,302 to $18,431).

**Conclusion:**

Total cost of care takes into account both the intensity of the treatment as well as the duration of the hospital stay. Thus, policy makers and health systems can use cost of care as a proxy for severity, especially when looking at the disparities among different race and ethnicity groups.

## Introduction

After a decade of moderate healthcare spending growth in the United States (US), a significant escalation occurred in 2020 [1, 2]. This surge in healthcare spending, largely attributed to the COVID-19 pandemic, resulted in per capita spending increasing by 12.3% between 2019 and 2021. This large increase in healthcare spending is partially attributed to COVID-19 hospitalization costs, estimated at $24,173 per patient, over 80% higher than the average cost of hospital stays overall prior to the pandemic [3, 4]. There remains an urgent need to identify factors linked to COVID-19 severity and the associated hospitalization costs. Public health officials and healthcare systems need this information to tailor programs for future infectious disease outbreaks to ensure patients receive appropriate care before their disease advances to the most severe levels.

Numerous studies have examined racial/ethnic disparities in COVID-19 hospital outcomes, including intensive care unit (ICU) utilization, intubation, hospital length of stay (LOS), and in-hospital mortality [5–9]. However, these individual outcome measures overlap and may not provide a comprehensive understanding of COVID-19 disparities in the hospital setting. Cost of care takes into account resource intensity and hospitalization duration, capturing LOS, ICU use, intubation, and death in a single measure, serving as a proxy for COVID-19 severity. Therefore, using total hospital cost of care to assess disparities in severe COVID-19 patients may shed new light for policymakers and healthcare providers. From an economic perspective, total cost of care is one practical metric for healthcare administrators and policymakers to evaluate COVID-19 disparities among hospitalized patients. While a few studies have reported COVID-19 hospitalization costs by patient race and ethnicity, they lacked detailed information about the underlying differences in resources and cost components contributing to these differences [10–12].

To address limitations of prior research, the study aims were to provide a detailed description of the hospital costs of COVID-19 based on individual resources during the hospital stay and standardized costs that do not rely on inflation adjustment, and therefore, more accurately capture treatment intensity, and to evaluate the extent to which hospital total cost of care for patients with COVID-19 differs by race and ethnicity. In the two studies that compared adjusted COVID-19 hospitalization costs by race/ethnicity, Hispanic patients had significantly higher costs compared to White or non-Hispanic patients, while results were inconsistent regarding costs between Black and White patients, with Tsai et al. reporting $3,012 higher costs for Black patients and Ohsfeldt et al. reporting similar costs for the two groups [11, 12]. Furthermore, we speculated that differences in underlying comorbidities among racial/ethnic groups and other patient and neighborhood characteristics would account for differences in costs. Therefore, we hypothesized the following: (1) non-Hispanic Black and Hispanic patients have higher unadjusted hospital costs compared to non-Hispanic White patients; and (2) after adjusting for patient and neighborhood characteristics, racial and ethnic differences in hospital costs are mitigated.

## Materials and methods

### Data sources and population

This retrospective observational study used data collected from the electronic medical record (EMR) and cost accounting system of Rush University Medical Center (RUMC), a large academic medical center on the West Side of Chicago, Illinois, with the third largest number of general medical-surgical and ICU beds of acute care hospitals in the metropolitan area. The sample included patients with confirmed SARS-CoV-2 infection or diagnosed with COVID-19 who were discharged between March 12, 2020, and April 30, 2021. The analysis was limited to patients hospitalized for COVID-19 based on the Centers for Disease Control and Prevention ICD-10 Guidelines for Coding and Reporting, encompassing the following diagnosis codes: principal ICD-10 diagnosis code U07.1, principal diagnosis code A41.89 with a secondary diagnosis of U07.1, and for discharges prior to April 1, 2020, principal diagnosis codes B97.29, J12.89, J20.8, J22, J40, J80, and J98.8, with a secondary diagnosis code of U07.1 [13, 14]. This study was approved as exempt by the Rush University Medical Center in accordance with US Code of Federal Regulations 45 CFR 46.104 (d)(4)(iii). Because data were retrospective, informed consent was waived. Data were retrieved between March 15, 2021, and July 22, 2021. Medical record number was used for linking data files and was excluded from the analysis. Subject names were not collected.

Patient-level data were obtained from the organization’s EMR. Patients were excluded if their charts were missing race, ethnicity, marital status, or home address. Since this study focused on the cost of the entire inpatient stay, patients who were transferred into RUMC were excluded. The number of patients excluded due to missing data was small (n = 117, 6%). Neighborhood socioeconomic measures at the census tract level were retrieved from the 2015–2019 five-year data file of the American Community Survey (ACS) of the US Census Bureau [15].

### Cost of care and length of stay calculations

Hospital cost data were obtained from the organization’s accounting system, reflecting the actual cost of delivering care from the hospital’s perspective. Hospital cost was calculated using a previously validated bottom-up approach, where each resource used and the associated per-unit cost were collected from the accounting system [16–18]. A standardized list of costs was created by calculating the average cost per resource to account for potential changes in per-unit costs during the study period. The quantity of each resource used was multiplied by its standardized cost to calculate the resource-level cost. Resources were classified into 11 components: ICU, general acute care, pharmacy, respiratory, laboratory and pathology, diagnostic services, dialysis, therapies, extracorporeal membrane oxygenation (ECMO), ED, and other). The resource-level costs were summed within each component to calculate the total component cost. Total hospital cost was calculated by summing costs across cost components. Hospital LOS was calculated by subtracting the patient’s discharge date from the admission date. For patients who died during the hospital stay, the death date was subtracted from the admission date.

### Patient characteristics

Patient sociodemographic characteristics included age, sex, marital status (married, not married), and primary payer (commercial insurers, Medicare, Medicaid, uninsured). During the study time period, the hospital’s system for recoding race and ethnicity was limited to one field for race and one field for ethnicity, limiting detailed information capture for multiple racial categories. As a result, we classified patients as Hispanic if they indicated Hispanic ethnicity, regardless of their racial category. For non-Hispanic patients, racial categories determined classification into non-Hispanic Black (Black), Non-Hispanic White (White) or other races. Other racial groups were excluded due to the small number of observations. Preferred language was classified as English or non-English, based on the patient’s self-reported language preference recorded in the EMR. The only non-English language reported by Hispanic patients was Spanish. Distance between the patient’s home residence and RUMC was included as a proxy for the ability to select the hospital for care and was measured in average driving time (minutes) per Google Maps.

Chronic conditions associated with higher COVID-19 severity were determined based on secondary ICD-10 diagnosis codes using the Clinical Classifications Software Refined algorithm [19]. Chronic conditions included chronic obstructive pulmonary disease (COPD), diabetes, heart disease, kidney disease, and obesity [20]. COVID-19 illness severity measures included ICU admission during the hospitalization (yes/no), direct admission from the ED to the ICU (yes/no), intubation (yes/no), and in-hospital death (yes/no).

### Neighborhood socioeconomic characteristics

Patients’ home addresses were converted to latitude and longitude, then geocoded to the census tract level, representing the patient neighborhood of home residence. Neighborhood socioeconomic characteristics related to COVID-19 risk were obtained from the ACS by census tract and included percent of workers classified as essential workers [21], percent of the population that was uninsured, percent of households receiving food assistance or Supplemental Nutrition Assistance Program (SNAP) benefits, percent of the population residing in an overcrowded household (>1.01 persons per room in housing unit), and whether the patient lived in a neighborhood with at least 30% of the population living below the federal poverty level (concentrated poverty) (S1 Table).

### Statistical analysis

We calculated frequencies and central measures for each variable by race/ethnicity and tested associations using χ^2^ tests and Mann Whitney U tests or Wilcoxon rank sum tests. Unadjusted mean differences in the total cost of care between racial/ethnic groups were calculated, along with 95% confidence intervals using 10,000 bootstrap samples with replacement to account for the skewed distribution of costs.

A generalized linear model (GLM) was used to evaluate the association between race/ethnicity and total cost, controlling for patient sociodemographic characteristics, average travel time to hospital, admission month and year, presence of chronic conditions, and neighborhood socioeconomic characteristics. Robust standard errors clustered at the census tract level were used. The link function for the GLM was determined using the Pregibon link test (log), and the distribution family was selected using the modified Parks test (inverse gaussian). To quantify the effect of race/ethnicity, we computed average marginal effects (AMEs) using recycled predictions from the regression model, representing the absolute change in adjusted cost between racial/ethnic groups [22, 23]. For example, to calculate the AME of Black versus White race, we computed the difference in adjusted cost for all patients if they were Black versus White, holding all covariates at their observed values. All p-values < 0.05 were considered statistically significant. Analyses were conducted using SAS version 9.4 (Cary, NC) and Stata version 17 (College Station, TX).

Two secondary analyses were conducted. First, we classified Hispanic patients by preferred language (English versus Spanish) then examined the association between race/ethnicity and total cost for Black, White, Hispanic with English language preference, and Hispanic with Spanish language preference groups. In another secondary analysis, we stratified patients by ICU admission during hospitalization to assess whether cost differences by race/ethnicity were attributed to higher COVID severity. An additional model for patients admitted to the ICU included direct ICU admission and intubation as covariates to further control for COVID-19 severity. Both sets of secondary analyses used GLMs to predict AMEs using the approach described for the main analysis, with GLMs estimated with a log link function and gamma distribution.

## Results

### Sample characteristics

Of the 1,853 patients included in the study, 773 (42%) were Black, 811 (44%) Hispanic, and 269 (14%) White (Table 1). Twenty-eight percent of Black patients had Medicaid coverage compared to 20% of Hispanic and 13% of White patients. A larger percentage of Hispanic patients (18%) were uninsured compared to Black (2%) and White (3%) patients. More than half (54%) of Hispanic patients reported Spanish as their preferred language. Black patients were most likely to have COPD, heart disease, kidney disease, and obesity, whereas Hispanic patients were most likely to have diabetes. Neighborhood characteristics differed across racial/ethnic groups, with Hispanic patients residing in neighborhoods with the largest proportions of essential workers, uninsured individuals, and residents living in crowded housing. Overall, 43% of Black patients resided in neighborhoods with concentrated poverty, compared to 13% of Hispanic and 3% of White patients.

**Table 1:** Description of the sample by race/ethnicity, N = 1853.

	Black N = 773 (42%)	Hispanic N = 811 (44%)	White N = 269 (14%)	p-value
Age, Med (IQR)	61 (48, 70)	57 (45, 68)	65 (51, 76)	< .001
Male, n (%)	336 (43.5)	461 (56.8)	143 (53.2)	< .001
Married, n (%)	185 (23.9)	451 (55.6)	141 (52.4)	< .001
Primary payer, n (%)				< .001
Commercial	183 (23.7)	249 (30.7)	90 (33.5)	
Medicare	361 (46.7)	259 (31.9)	137 (50.9)	
Medicaid	214 (27.7)	161 (19.9)	35 (13.0)	
Uninsured	15 (1.9)	142 (17.5)	7 (2.6)	
Preferred language, n (%)				< .001
English	769 (99.5)	372 (45.9)	247 (91.8)	
Non-English	4 (0.5)	439 (54.1)	22 (8.2)	
Driving time (minutes), Med (IQR)	15.5 (9.4, 22.8)	18.3 (13.7, 23.6)	22.1 (14.7, 34.4)	< .001
Chronic conditions				
COPD, n (%)	113 (14.6)	39 (4.8)	30 (11.2)	< .001
Diabetes, n (%)	320 (41.4)	401 (49.5)	86 (32.0)	< .001
Heart disease, n (%)	637 (82.4)	504 (62.2)	203 (75.5)	< .001
Kidney disease, n (%)	301 (38.9)	137 (16.9)	66 (23.4)	< .001
Obesity, n (%)	502 (64.9)	456 (56.2)	126 (46.8)	< .001
COVID-19 Severity				
ICU stay, n (%)	243 (31.4)	325 (40.1)	87 (32.0)	< .001
Directly admitted to ICU, n (%)	155 (20.1)	198 (24.4)	46 (17.1)	.017
Intubation, n (%)	82 (10.6)	131 (16.2)	23 (8.6)	< .001
Hospital death, n (%)	40 (5.2)	62 (7.6)	15 (5.6)	.112
Neighborhood Characteristics				
% essential workers, Med (IQR)	26.5 (21.6, 32.9)	27.2 (23.4, 31.6)	24.2 (19.3, 29.6)	< .001
% uninsured, Med (IQR)	8.2 (5.4, 11.8)	14.0 (9.6, 18.5)	5.6 (3.1, 10.0)	< .001
% households receiving SNAP benefits, Med (IQR)	36.5 (22.8, 45.5)	21.1 (13.7, 29.6)	9.6 (3.6, 15.3)	< .001
% households in overcrowded housing (>1 person/room), Med (IQR)	2.5 (1.1, 3.9)	4.9 (2.5, 8.2)	2.0 (0.7, 3.7)	< .001
Concentrated poverty (30%+ below federal poverty level), n (%)	332 (43.0)	109 (13.4)	9 (3.4)	< .001

There were significant racial/ethnic differences in the proportion of patients with an ICU admission, with 40% of Hispanic patients admitted to the ICU, compared to 31% of Black and 32% of White patients (p < .001). However, there was no difference in in-hospital death by race/ethnicity (Table 1).

### Unadjusted total cost of care and length of stay by patient race/ethnicity

The largest portions of costs were attributed to the ICU and general acute care, followed by pharmacy and respiratory care. Hispanic patients with Spanish language preference had the highest costs in most cost components, with the greatest absolute difference in ICU costs (S1 Fig and S2 Table). Unadjusted mean cost of care differed by patient sex, marital status, driving time and distance, and several neighborhood characteristics (S3 Table).

Hispanic patients had the highest unadjusted mean cost of care (Table 2), with $9,204 (95% confidence interval (CI): $4,339 to $14,068) and $6,287 (95% CI: $144 to $12,430) higher costs, compared to Black and White patients, respectively. There was no statistically significant difference between Black and White patients’ unadjusted costs. These differences roughly correspond to the unadjusted mean differences in LOS by race/ethnicity, with Hispanic patients staying approximately two days longer than Black and White patients (S4 Table). Hispanic patients who preferred Spanish had a $11,478 higher unadjusted mean total cost of care than Hispanic patients who preferred English (S5 Table) and LOS of more than three days longer on average compared to other patients. Unadjusted mean total cost and LOS were similar for Hispanic patients who preferred English, Black and White patients.

**Table 2:** Unadjusted mean total cost of care and difference in costs by race/ethnicity.

	All Patients	White	Black	Hispanic
Total Cost, Mean (sd)	26,254 (48,901)	24,719 (37,096)	21,803 (34,446)	31,006 (61,960)
Difference, Relative to White, Mean (95% CI)	--	--	-2,916 (-7,957 to 2,124)	6,287 (144 to 12,430)*
Difference, Relative to Black, Mean (95% CI)	--	--	--	9,204 (4,339, 14,068)*

Notes: 95% CI of differences in mean costs estimated from a nonparametric bootstrap procedure with 10,000 resamples.

*p < .05.

### Association of patient race/ethnicity and total cost of care

**Fig 1:**
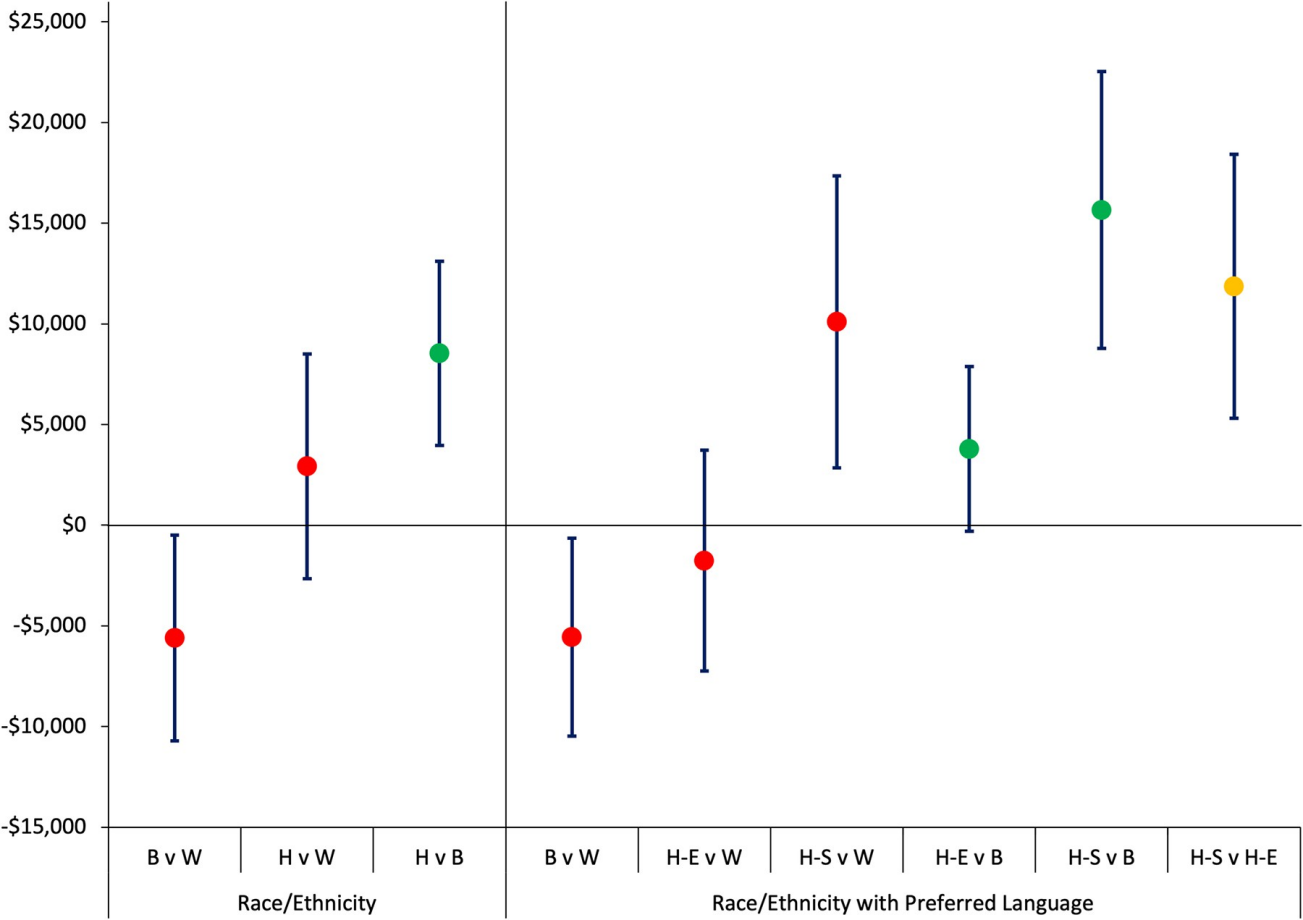
Average marginal effects of race/ethnicity on total cost of care and 95% confidence intervals. B = Black; H-E = Hispanic, English Preferred Language; H-S = Hispanic, Spanish Preferred Language, W = White. Dots represent predicted average marginal cost and lines represent 95% confidence intervals. Red circle Reference = White; Green circle Reference = Black; Yellow circle Reference = Hispanic, English Preferred Language. AMEs were estimated using a generalized linear model with a log link function and inverse Gaussian distribution. Robust standard errors clustered at the census tract level were computed. *p < .05. Model patient demographic characteristics (race/ethnicity, age, sex, marital status), primary payer, average travel time from home residence to hospital, presence of chronic conditions, neighborhood socioeconomic factors (proportion of workers classified as essential, proportion of population that is uninsured, proportion of households receiving SNAP benefits, proportion of housing units that are overcrowded, and neighborhood with high concentrated poverty), and month-year of admission.

After adjusting for patient and neighborhood characteristics, total cost of care was still significantly higher for Hispanic compared to Black patients, with an AME of $8,539 (95% CI: $3,963 to $13,115), but not significantly different from White patients. Conversely, Black patients had significantly lower costs compared to White patients, with an AME of -$5,606 (Table 3 and Fig 1). Adjusted total cost of care was significantly higher for Hispanic patients who preferred Spanish compared to all other racial/ethnic groups (Table 4 and Fig 1): cost of care was $11,866 (95% CI: $5,302 to $18,431) higher compared to Hispanic patients who preferred English, $10,104 (95% CI: $2,849 to $17,358) higher compared to White patients, and $15,656 (95% CI: $8,788 to $22,524) higher compared to Black patients. Costs were not significantly different among Hispanic patients who preferred English, White and Black patients. Adjusted mean total cost of care is reported in S6 Table. Neighborhood characteristics were not significantly associated with total cost in either model, after adjusting for patient characteristics (results not shown).

**Table 3:** Average marginal effect estimates for total cost of care by race/ethnicity.

Racial/Ethnic Group Comparison	AME in USD	95% CI
Black versus White	-5,606*	-10,711 to -501
Hispanic versus White	2933	-2,646 to 8,512
Hispanic versus Black	8,539*	3,963 to 13,115

AME, average marginal effect. AMEs were estimated using a generalized linear model with a log link function and inverse Gaussian distribution. Robust standard errors clustered at the census tract level were computed. *p < .05. Model includes patient demographic characteristics (race/ethnicity, age, sex, marital status), primary payer, average travel time from home residence to hospital, presence of chronic conditions, neighborhood socioeconomic factors (proportion of workers classified as essential, proportion of population that is uninsured, proportion of households receiving SNAP benefits, proportion of housing units that are overcrowded, and neighborhood with high concentrated poverty), and month-year of admission.

**Table 4:** Average marginal effect estimates for total cost of care by race/ethnicity with language preference for Hispanic patients.

Racial/Ethnic Group Comparison	AME in USD	95% CI
Black versus White	-5,553*	-10,465 to -640
Hispanic-English Preference versus White	-1,763	-7247 to 3,721
Hispanic-English Preference versus Black	3,790	-310 to 7,890
Hispanic-Spanish Preference versus White	10,104*	2,849 to 17,358
Hispanic-Spanish Preference versus Black	15,656*	8,788 to 22,524
Hispanic-Spanish Preference versus Hispanic-English Preference	11,866*	5,302 to 18.431

AME, average marginal effect. AMEs were estimated using a generalized linear model with a log link function and inverse Gaussian distribution. Robust standard errors clustered at the census tract level were computed. *p < .05. Model includes patient demographic characteristics (race/ethnicity, age, sex, marital status), primary payer, average travel time from home residence to hospital, presence of chronic conditions, neighborhood socioeconomic factors (proportion of workers classified as essential, proportion of population that is uninsured, proportion of households receiving SNAP benefits, proportion of housing units that are overcrowded, and neighborhood with high concentrated poverty), and month-year of admission.

### COVID-19 severity and total cost of care

To determine the extent to which cost differences were driven by higher COVID severity, we performed a secondary analysis that stratified patients by ICU utilization. For patients without ICU utilization, we found no significant difference in costs between any racial/ethnic groups (S2 Fig). Before controlling for COVID severity in the model limited to patients admitted to the ICU, Hispanic patients who preferred Spanish had significantly higher costs compared to Black patients and Hispanic patients who preferred English. However, after controlling for COVID-19 severity (direct ICU admission from the ED, intubation), we found no significant difference in costs among Hispanic patients preferring Spanish and other patients (S2 Fig).

## Discussion

This study compared the total hospital cost of care among Black, Hispanic, and White patients hospitalized for COVID-19. After adjusting for patient and neighborhood characteristics, Black patients had significantly lower costs compared to White patients, and although Hispanic patients had significantly higher costs overall, the higher cost was attributed to the subset of Hispanic patients who preferred Spanish, with no significant difference in costs among Hispanic patients who preferred English, Black and White patients. Our results can be compared with those of two studies that reported hospitalization costs for COVID-19 by race/ethnicity using data from more than 800 hospitals participating in the Premier Healthcare Database. Ohsfeldt et al. and Shrestha et al. reported either similar or modestly lower costs for Black compared to White patients and higher costs for Hispanic versus non-Hispanic patients [4, 11]. The similarities and differences are easier to understand when converted to relative costs, i.e., ratio of two costs. We found substantially larger relative differences in costs between Black and White patients (relative costs = 0.75 in our study versus 0.99 in Ohsfeldt et al. and 0.95 in Shrestha et al.), and although the cost differences between Hispanic and White patients were not significant in our study, the relative differences were similar to Ohsfeldt et al. (relative costs for Hispanic versus White = 1.12 in Ohsfeldt et al. and our study) but smaller than in Shrestha et al. (relative costs = 1.24). Our findings suggest that comparisons of costs across studies for Hispanic patients may be confounded by unobserved heterogeneity that is related to language preference, and we speculate language preference may be an important variable to include in future studies [24].

We found that Black patients had more comorbidities than White patients but a significantly lower cost of care, contrary to our original hypothesis. One possible explanation for this difference is that more Black patients may have been admitted for precautionary reasons due to a high comorbidity burden but with lower COVID severity compared to White patients. These results are consistent with findings from a large, national sample of COVID-19 hospital outcomes in the US that examined disparities by race and gender identity [9]. Pal et al. reported higher incidence of comorbidities, including hypertension, diabetes, renal failure, and obesity in Black men and women compared to White men and women hospitalized for COVID-19, however, both Black men and Black women were less likely to die or be intubated while hospitalized, compared to White men in adjusted analyses [9]. Although we did not observe higher rates of ICU use, intubation or in-hospital death for Black patients compared to other patients, future work should examine whether there were disparities in outcomes after hospital discharge, such as increased risk of hospital readmission, poor health, or mortality.

While we did not find differences in costs between Hispanic and White patients overall, Hispanic patients who preferred Spanish had nearly $12,000 higher costs compared to Hispanic patients who preferred English and $10,000 higher costs compared to White patients (who preferred English), suggesting that language may be an important mediator. Other studies and our secondary analysis of costs stratified by ICU use corroborate this speculation. In a study of hospitalization outcomes for Hispanic versus non-Hispanic patients in Texas, Velasco et al. reported the odds of ICU care was 1.47 times higher for Hispanic non-English speaking patients compared to White patients, with no significant difference between Hispanic English-speaking patients and White patients [25]. Additionally, in a study using similar data from our own institution, Hispanic patients with COVID-19 who used interpreter services were at greater risk for in-hospital death compared to Hispanic patients who did not use an interpreter [24]. Other studies examining the role of acculturation in healthcare utilization for Hispanic patients have demonstrated significant differences in utilization by level of acculturation. In a study of COVID-19 hospitalizations in 12 Minnesota hospitals, Ingraham et al. found that Hispanic non-English-speaking adults were nearly four times as likely to be admitted to the hospital as Hispanic English-speaking adults after testing positive for COVID-19, suggesting higher severity of illness for non-English speaking adults [26]. In our study, preferred language was likely a proxy for other factors that contributed to higher illness severity for hospitalized patients, such as long-term lack of health insurance coverage, unfamiliarity with the US healthcare system and US hospitals, education, healthcare literacy, fear of US hospitals, and economic concerns, such forgone earnings while in the hospital and out-of-pocket costs. It is imperative that barriers to healthcare use by non-English speaking patients are pinpointed, so that interventions can be developed to facilitate timely care for these patients, particularly with infectious disease outbreaks.

This study has several limitations worth noting. Although Pal et al. found large differences in outcomes by gender identity within racial and ethnic groups [9], our sample size was not large enough to stratify analyses by race/ethnicity and sex (we did not have information on gender identity). A future study with a larger sample size should examine whether differences in hospital costs among racial/ethnic groups exist after stratifying by sex and/or gender identity. Additionally, we had detailed costs from only one academic medical center, and the absolute dollar amounts may differ from other hospitals. However, with standardized costs from a single institution, we have removed unobserved variation in cost structures and inflation that are inherent in multi-hospital studies. Finally, our study was limited to hospitalized patients, thereby censoring less severe patients from our data set, and differences in ED and primary care use for COVID-19 symptoms may explain differences in COVID-19 severity by race and ethnicity that we observed for hospitalized patients [27, 28].

## Conclusion

COVID-19 hospitalization costs and treatments differed significantly for Hispanic patients based on their language preference, suggesting an important but rarely considered mediator between race/ethnicity, COVID-19 severity and hospitalization costs. The higher hospitalization costs for Hispanic patients who preferred Spanish was attributed to greater ICU utilization. This study captured racial/ethnic differences in costs for patients needing hospital care that is, in general, more expensive than care in the ED or with a primary care physician.

## Supporting information

S1 Fig Unadjusted mean total cost of care by cost category and race/ethnicity with language preference for Hispanic patients. (TIF)

S2 Fig Average marginal effect of race/ethnicity on total cost of care and 95% confidence intervals, stratified by intensive care unit use. B = Black; H-E = Hispanic, English Preferred Language; H-S = Hispanic, Spanish Preferred Language, W = White. Dots represent predicted average marginal cost and lines represent 95% confidence intervals. Red circle Reference = White; Green circle Reference = Black; Yellow circle Reference = Hispanic, English Preferred Language. AMEs were estimated using a generalized linear model with a log link function and gamma distribution. Robust standard errors clustered at the census tract level were computed. p < .05. *Models include patient demographic characteristics (race/ethnicity, age, sex, marital status), primary payer, average travel time from home residence to hospital, presence of chronic conditions, neighborhood socioeconomic factors (proportion of workers classified as essential, proportion of population that is uninsured, proportion of households receiving SNAP benefits, proportion of housing units that are overcrowded, and neighborhood with high concentrated poverty), and month-year of admission. **Model also includes direct ICU admission (yes/no) and intubation (yes/no) as covariates.
(TIF)

S1 Table Neighborhood socioeconomic characteristics collected from the 2015–2019 5-Year data file of the American Community Survey. (PDF)

S2 Table Unadjusted mean cost by cost category and race/ethnicity in US dollars. (PDF)

S3 Table Unadjusted mean total cost of care by socio-demographic and neighborhood characteristics. ^a^ Significantly different, p < .05; ^b^ Significantly different from Quartile 1, p < .05; ^c^ Significantly different from Quartile 2, p < .05; ^d^ Significantly different from Quartile 3, p < .05; ^e^ Significantly different from Quartile 4, p < .05.
(PDF)

S4 Table Unadjusted mean length of stay and length of stay differences by race/ethnicity. 95% CI of differences in mean costs estimated from a nonparametric bootstrap procedure with 10,000 resamples. *p < .05.
(PDF)

S5 Table Unadjusted mean total cost of care and length of stay and differences by race/ethnicity with preferred language for Hispanic patients. 95% CI of differences in mean costs and length of stay estimated from nonparametric bootstrap procedures with 10,000 resamples.
(PDF)

S6 Table Predicted total cost of care (predictive margin) by race/ethnicity. Models control for patient demographic characteristics (race/ethnicity, age, sex, marital status), primary payer, average travel time from home residence to hospital, presence of chronic conditions, neighborhood socioeconomic factors (proportion of workers classified as essential, proportion of population that is uninsured, proportion of households receiving SNAP benefits, proportion of housing units that are overcrowded, and neighborhood with high concentrated poverty), and month-year of admission.
(PDF)
