# Supplementary figures and images for: Racial and ethnic disparities in COVID-19 hospital cost of care
Source: PLoS One. 2024 Oct 14;19(10):e0309159. doi: 10.1371/journal.pone.0309159 (PMC11472913; doi:10.1371/journal.pone.0309159)

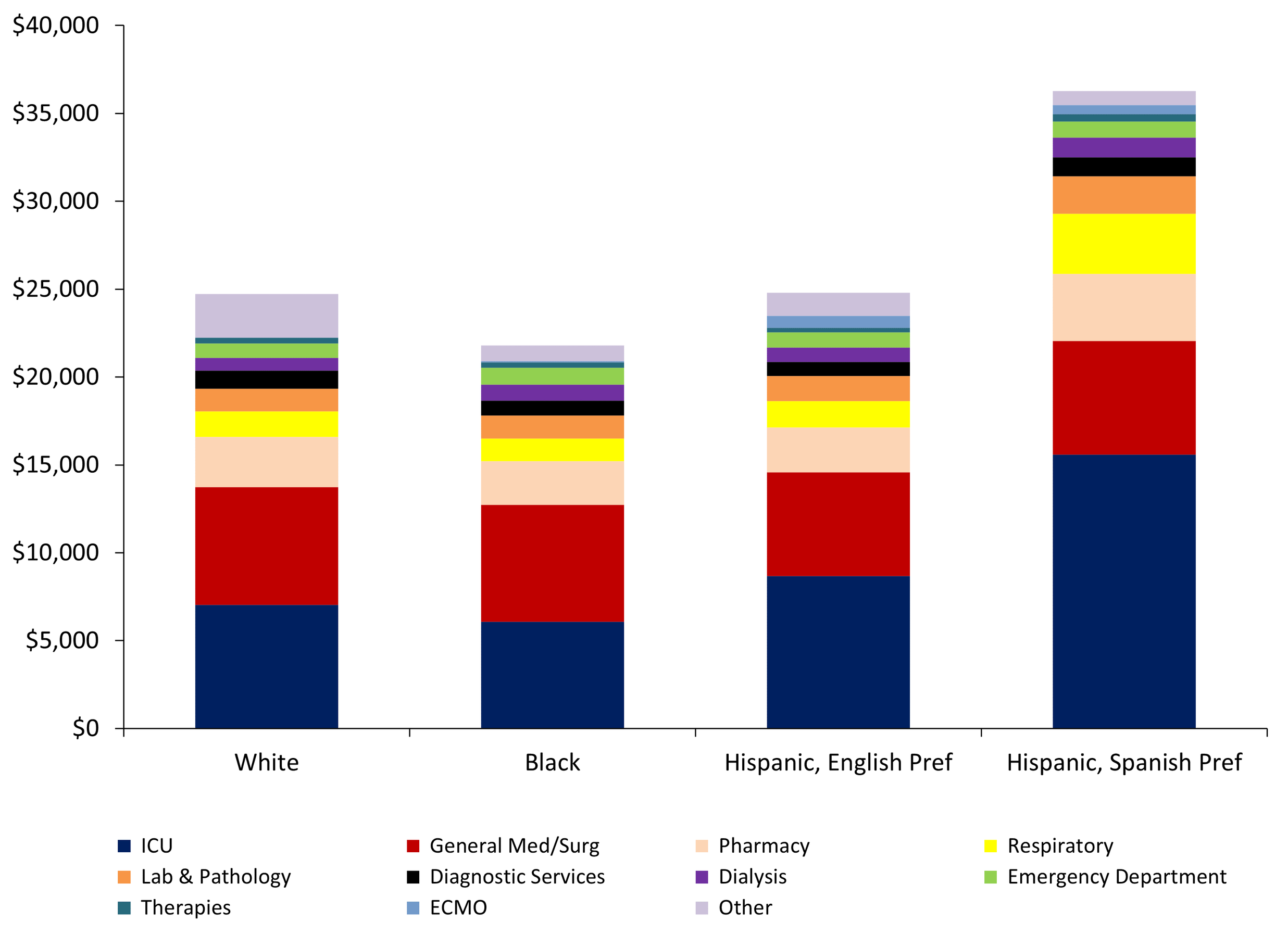

Supplement: S1 Fig — (TIF) [file pone.0309159.s001.tif]

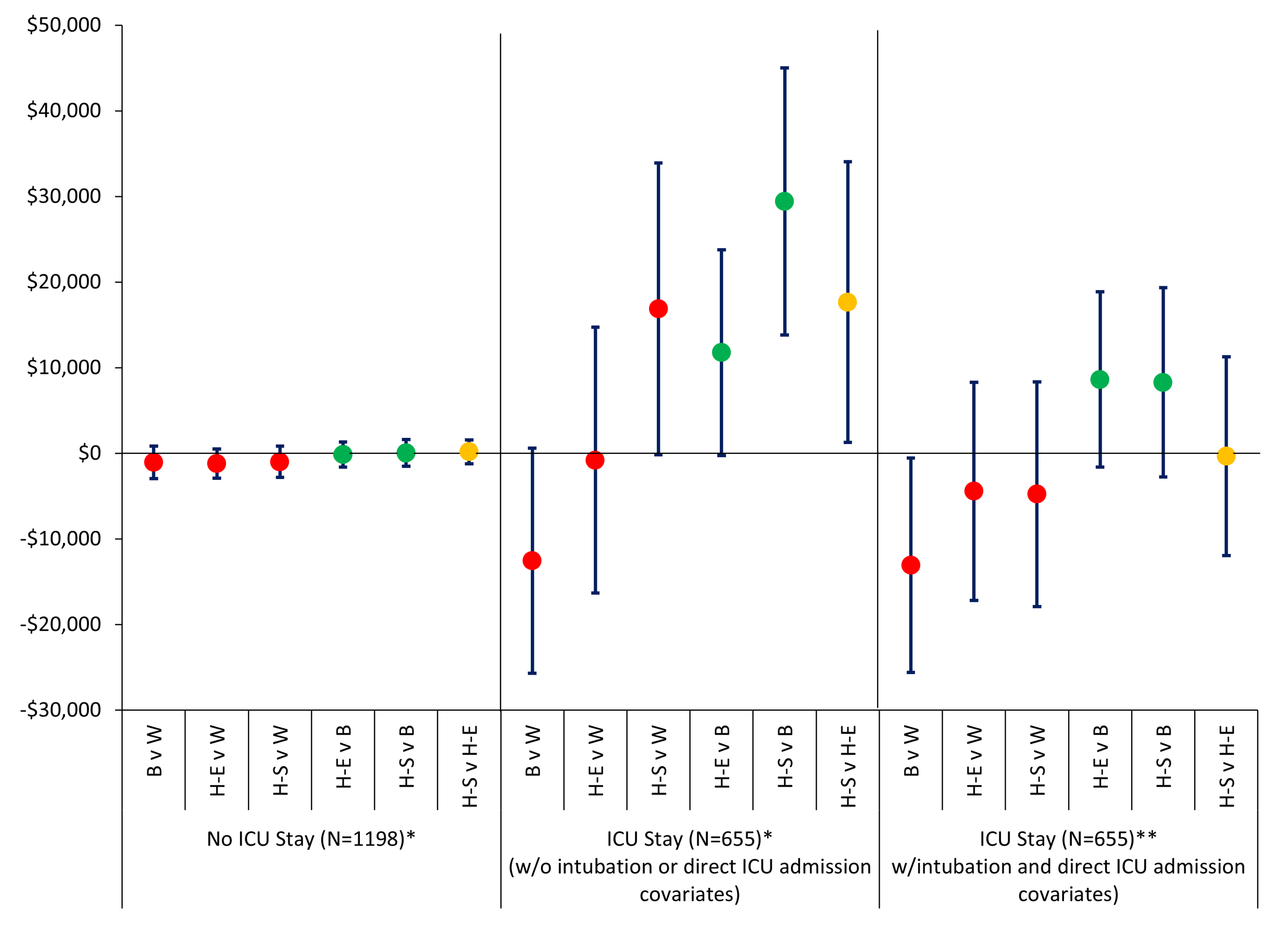

Supplement: S2 Fig — B = Black; H-E = Hispanic, English Preferred Language; H-S = Hispanic, Spanish Preferred Language, W = White. Dots represent predicted average marginal cost and lines represent 95% confidence intervals. Red circle Reference = White; Green circle Reference = Black; Yellow circle Reference = Hispanic, English Preferred Language. AMEs were estimated using a generalized linear model with a log link function and gamma distribution. Robust standard errors clustered at the census tract level were computed. p < .05. *Models include patient demographic characteristics (race/ethnicity, age, sex, marital status), primary payer, average travel time from home residence to hospital, presence of chronic conditions, neighborhood socioeconomic factors (proportion of workers classified as essential, proportion of population that is uninsured, proportion of households receiving SNAP benefits, proportion of housing units that are overcrowded, and neighborhood with high concentrated poverty), and month-year of admission. **Model also includes direct ICU admission (yes/no) and intubation (yes/no) as covariates. (TIF) [file pone.0309159.s002.tif]
