# Supplementary material for: Racial and ethnic disparities in COVID-19 hospital cost of care
Source: PLoS One. 2024 Oct 14;19(10):e0309159. doi: 10.1371/journal.pone.0309159 (PMC11472913; doi:10.1371/journal.pone.0309159)
Supplement: S1 Table — (PDF) [file pone.0309159.s003.pdf]

Supplemental Table 1. Neighborhood Socioeconomic Characteristics Collected from the 2015-2019 5-Year Data File of the American Community Survey

Variable	Description
Percent of workers that are essential workers	Total number of workers in construction, food industry, healthcare, community and social services, transportation and protective services divided by the total number of workers
Percent of population that is uninsured	Total population that is uninsured divided by total population
Percent of households receiving SNAP benefits	Total number of households receiving food stamps or SNAP benefits divided by total number of households
Percent of units that are overcrowded	Total number of housing units with 1.01 or more people per room divided by the total number of housing units
Concentrated poverty	Coded as 1 if > 30% of population is living below the federal poverty line, and coded as 0 if ≤ 30% of population in census tract is living below the federal poverty level
