# Supplementary material for: Racial and ethnic disparities in COVID-19 hospital cost of care
Source: PLoS One. 2024 Oct 14;19(10):e0309159. doi: 10.1371/journal.pone.0309159 (PMC11472913; doi:10.1371/journal.pone.0309159)
Supplement: S2 Table — (PDF) [file pone.0309159.s004.pdf]

Supplemental Table 2. Unadjusted Mean Cost by Cost Category and Race/Ethnicity in US Dollars

Race/Ethnicity	ICU	General Acute Care	Pharmacy	Respiratory	Laboratory and Pathology	Diagnostic Services	Dialysis	Emergency Department	Therapies	ECMO	Other
White	7,029	6,718	2,850	1,452	1,296	1,024	730	810	340	-	2,471
Black	6,082	6,656	2,478	1,284	1,303	860	904	954	314	62	905
Hispanic, English Preferred Language	8,673	5,907	2,562	1,481	1,440	797	825	850	261	682	1,315
Hispanic, Spanish Preferred Language	15,585	6,481	3,818	3,398	2,144	1,088	1,111	924	408	512	802
