# Supplementary material for: Racial and ethnic disparities in COVID-19 hospital cost of care
Source: PLoS One. 2024 Oct 14;19(10):e0309159. doi: 10.1371/journal.pone.0309159 (PMC11472913; doi:10.1371/journal.pone.0309159)
Supplement: S3 Table — a Significantly different, p < .05; b Significantly different from Quartile 1, p < .05; c Significantly different from Quartile 2, p < .05; d Significantly different from Quartile 3, p < .05; e Significantly different from Quartile 4, p < .05. (PDF) [file pone.0309159.s005.pdf]

Supplemental Table 3. Unadjusted Mean Total Cost of Care by Socio-Demographic and Neighborhood Characteristics

Variable	Mean (sd)
Primary payer	
Commercial	24,759 (47,881)
Medicare	26,299 (37,009)
Medicaid	28,137 (68,270)
Uninsured	26,102 (42,170)
Sex	
Male	29,731 (58,313) [a]
Female	22,675 (41,196) [a]
Marital status	
Married	22,998 (36,629) [a]
Not married	30,763 (61,752) [a]
Arrival mode	
Ambulance	26,154 (50,357)
Not ambulance	26,920 (37,882)
Rush PCP visit in prior 24 months	
Yes	27,428 (41,196)
No	25,092 (55,485)
Has any primary care physician	
Yes	26,044 (48,603)
No	27,460 (51,238)
Driving time, minutes	
Quartile 1 (shortest)	21,958 (34,773) [c]
Quartile 2	28,932 (46,600) [b]
Quartile 3	27,792 (61,749)
Quartile 4 (longest)	26,440 (48,611)
Driving distance, miles	

Variable	Mean (sd)
Quartile 1 (shortest)	23,519 (36,436) [c]
Quartile 2	28,564 (62,324) [b]
Quartile 3	29,308 (55,666)
Quartile 4 (longest)	23,475 (34,125)
% essential workers	
Quartile 1 (lowest)	26,169 (60,356)
Quartile 2	23,223 (24,696)
Quartile 3	28,275 (50,347)
Quartile 4 (highest)	27,381 (46,553)
% uninsured	
Quartile 1 (lowest)	22,401 (31,821) [e]
Quartile 2	23,602 (36,947) [e]
Quartile 3	26,344 (44,874)
Quartile 4 (highest)	32,602 (71,416) [b,c]
% households receiving SNAP benefits	
Quartile 1 (lowest)	23,108 (33,896)
Quartile 2	29,013 (51,852)
Quartile 3	27,518 (63,749)
Quartile 4 (highest)	25,290 (40,407)
% overcrowded households (>1 person/room)	
Quartile 1 (lowest)	21,370 (30,748) [d,e]
Quartile 2	26,168 (59,727)
Quartile 3	26,831 (50,463) [b]
Quartile 4 (highest)	30,753 (49,896) [b]
Concentrated poverty (30%+ below FPL)	
Not concentrated	27,310 (52,643) [a]
Concentrated	22,963 (34,581) [a]

[a] Significantly different, $p<.05$; [b] Significantly different from Quartile 1, $p<.05$; [c] Significantly different from Quartile 2, $p<.05$; [d] Significantly different from Quartile 3, $p<.05$; [e] Significantly different from Quartile 4, $p<.05$
