# Supplementary material for: Racial and ethnic disparities in COVID-19 hospital cost of care
Source: PLoS One. 2024 Oct 14;19(10):e0309159. doi: 10.1371/journal.pone.0309159 (PMC11472913; doi:10.1371/journal.pone.0309159)
Supplement: S4 Table — 95% CI of differences in mean costs estimated from a nonparametric bootstrap procedure with 10,000 resamples. *p < .05. (PDF) [file pone.0309159.s006.pdf]

Supplemental Table 4. Unadjusted Mean Length of Stay and Length of Stay Differences by Race/Ethnicity

	White	Black	Hispanic
Length of Stay, Mean (sd)	7.7 (8.8)	7.4 (9.2)	9.5 (12.2)
Difference, Relative to White, Mean (95% CI)	--	-0.3 (-1.5 to 0.9)	1.8 (0.4 to 3.1)
Difference, Relative to Black, Mean (95% CI)	--	--	2.1 (1.0 to 3.1)

Notes: 95% CI of differences in mean costs estimated from a nonparametric bootstrap procedure with 10,000 resamples. *p<0.05.
