# Supplementary material for: Racial and ethnic disparities in COVID-19 hospital cost of care
Source: PLoS One. 2024 Oct 14;19(10):e0309159. doi: 10.1371/journal.pone.0309159 (PMC11472913; doi:10.1371/journal.pone.0309159)
Supplement: S5 Table — 95% CI of differences in mean costs and length of stay estimated from nonparametric bootstrap procedures with 10,000 resamples. (PDF) [file pone.0309159.s007.pdf]

Supplemental Table 5. Unadjusted Mean Total Cost of Care and Length of Stay and Differences by Race/Ethnicity with Preferred Language for Hispanic Patients

	White	Black	Hispanic, English Preferred Language	Hispanic, Spanish Preferred Language
Total Cost, Mean (sd), dollars	24,719 (37,096)	21,803 (34,446)	24,793 (53,044)	36,271 (68,233)
Difference, Relative to White, Mean (95% CI)	--	-2,916 (-7,957 to 2,124)	74 (-6,806 to 6,954)	11,552 (3,785 to 19,319)
Difference, Relative to Black, Mean (95% CI)	--	--	2,991 (-2,938 to 8,919)	14,468 (7,612 to 21,324)
Difference, Relative to Hispanic – English Pref, Mean (95% CI)	--	--	--	11,478 (3,149 to 19,807)
Length of Stay, Mean (sd)	7.7 (8.8)	7.4 (9.2)	7.8 (10.6)	10.9 (13.2)
Difference, Relative to White, Mean (95% CI)	--	-0.3 (-1.5 to 0.9)	0.1 (-1.4 to 1.6)	3.2 (1.6 to 4.9)
Difference, Relative to Black, Mean (95% CI)	--	--	0.4 (-0.9 to 1.6)	3.5 (2.1 to 4.9)
Difference, Relative to Hispanic-English Pref, Mean (95% CI)	--	--	--	3.2 (1.5 to 4.8)

Notes: 95% CI of differences in mean costs and length of stay estimated from nonparametric bootstrap procedures with 10,000 resamples
