# Supplementary material for: Racial and ethnic disparities in COVID-19 hospital cost of care
Source: PLoS One. 2024 Oct 14;19(10):e0309159. doi: 10.1371/journal.pone.0309159 (PMC11472913; doi:10.1371/journal.pone.0309159)
Supplement: S6 Table — Models control for patient demographic characteristics (race/ethnicity, age, sex, marital status), primary payer, average travel time from home residence to hospital, presence of chronic conditions, neighborhood socioeconomic factors (proportion of workers classified as essential, proportion of population that is uninsured, proportion of households receiving SNAP benefits, proportion of housing units that are overcrowded, and neighborhood with high concentrated poverty), and month-year of admission. (PDF) [file pone.0309159.s008.pdf]

Supplemental Table 6. Predicted Total Cost of Care (Predictive Margin) by Race/Ethnicity

	Adjusted Total Cost in USD	95% CI
Black	21,545	18,845 to 24,246
Hispanic	30,084	26,313 to 33,855
White	27,151	22,443 to 31,859
Black	21,026	18,321 to 23,732
Hispanic-English Preference	24,817	21,061 to 28,573
Hispanic-Spanish Preference	36,683	30,359 to 43,006
English	26,579	21,972 to 31,187

Models control for patient demographic characteristics (race/ethnicity, age, sex, marital status), primary payer, average travel time from home residence to hospital, presence of chronic conditions, neighborhood socioeconomic factors (proportion of workers classified as essential, proportion of population that is uninsured, proportion of households receiving SNAP benefits, proportion of housing units that are overcrowded, and neighborhood with high concentrated poverty), and month-year of admission.
